# Participation in the Long-Term Care Equality Index to Support Older LGBTQ+ Military Veterans

**DOI:** 10.1093/geroni/igaf122.1814

**Published:** 2025-12-31

**Authors:** Michelle Hilgeman, Sylvia Haigh, Jessica Wilkins, Elizabeth Western, Teddy Bishop, Jamie Key, David Hollingsworth, Whitney Mills

**Affiliations:** Tuscaloosa VA Medical Center, Tuscaloosa, Alabama, United States; VA Providence Healthcare System, Providence, Rhode Island, United States; Tuscaloosa VA Medical Center, Tuscaloosa, Alabama, United States; Tuscaloosa VA Medical Center, Tuscaloosa, Alabama, United States; Tuscaloosa VA Medical Center, Tuscaloosa, Alabama, United States; Tuscaloosa VA Medical Center, Tuscaloosa, Alabama, United States; Fielding Graduate University, Washington, District of Columbia, United States; Brown University, Providence, Rhode Island, United States

## Abstract

There are more than 500,000 older LGBTQ+ Veterans currently living in the United States. The US Veterans Health Administration (VHA) aspires to align care preferences to individualized goals and to offer high quality care to all Veterans. Consistent with these priorities, a growing number of VHA Community Living Centers (CLCs) have participated in the Long-Term Care Equality Index (LEI) developed by the Human Rights Foundation and the national Advocacy & Services for LGBTQ Elders group (SAGE). We used mixed methods to describe the formative evaluation process used to complete the LEI tool in 2023 and again in 2025 in a 150-bed CLC in the Deep South that specializes in long-term care, dementia care, mental health recovery, and hospice/palliative care. LGBTQ+ Veterans, CLC residents, clinicians, and administrators contributed to the LEI completion in 2023 resulting in a High Performer designation. Scores across all 4 domains of the LEI were maintained at 100% (foundational policies) or increased in demonstrated best practices from 2023 to 2025 (i.e., 60% to 73% in resident services/supports, 58% to 67% in employee benefits/policies, and 14% to 29% in resident/community engagement). Online trainings were completed by more than 50% of CLC staff (N = 212) with 59.1% (n = 127) completing an introductory course and 56.3% (n = 120) completing modules on sexual orientation or care for older gender diverse Veterans. In conclusion, the LEI offers an opportunity for residential communities to identify, measure, and share best practices within healthcare systems to improve care quality for all residents.

